# Report of Six Cases of Fracture of the Lower Extremity, Treated in the Surgical Wards of the Pennsylvania Hospital, after the Method of Suetin and Velpeau

**Published:** 1839-03-02

**Authors:** T. Harris

**Affiliations:** Attending Surgeon


### CLINICAL REPORTS.

*Report of six cases of fracture of the lower extremity, treated in the Surgical Wards of the Pennsylvania Hospital, after the method of Suetin and Velpeau.* Dr. T. Harris, Attending Surgeon.

[Reported by H. H. Smith, M. D., Resident Surgeon.]

In numbers twenty-one and twenty-three, of the Examiner for 1838, there appeared two articles on the Appareil Immobile, and its modifications in the treatment of fractures; as used by Suetin and Velpeau. As the advantages to be derived from this plan were very great, and as a large number of cases of this injury were constantly admitted into the Hospital, an early opportunity was sought for its application. The success of the first case was such as to cause its repetition in five others, all of which have done well and have gone far to prove its convenience to the patient, and its utility as a dressing for this very common class of accidents. The six cases, hereinafter reported, comprehend most of the patients labouring under this injury, who have been admitted since January, the only ones in which it has not been used being those which were deemed unfit, from the severe contusion of the limb consequent on the cause of fracture.

Case First.—*Fracture of both bones of the leg—Application of the apparatus seven days after the accident—Cure without deformity.*

George R——, æt. 34 years, a shoemaker by trade, and of temperate habits, was admitted into the Hospital, Dec. 25th 1838, with a fracture of the tibia, oblique at its lower third, and one of the fibula at its upper third caused by a fall upon the ice. The limb at first was placed in the fracture box, and evaporating lotions were used to reduce the inflammation, which was considerable. On the third of January, seven days after the accident, the immovable apparatus was applied in the following manner. A *washed* roller was smoothly applied, from the toes to the knee, cotton being placed along the spine of the tibia to prevent excoriation from the turns of the bandage, and also in the cavity, on each side of the tendo-achilles. This was well rubbed over with starch, made thick and smooth by being boiled for twenty minutes. A second roller was then applied from the knee down and also well covered with starch. Two pieces of binder's board cut to fit the sides of the leg and extend from below the knee to below the malleoli, were soaked in water until soft. They were then rubbed with starch, and applied to the leg over this, so as to surround it, except for the breadth of a finger on the front and back; small cuts being made at the lower end to cause it to fit the projection of the malleoli, and also at any other point where it bulged out. A third splint, made to fit the foot, and slit at the end so as to enable it to turn up behind the heel, was then applied to the foot, starched and secured by a third roller from the toes up. This was coated in like manner—a fourth applied over all, and the dressing completed by starch, which kept the whole smooth and tight without the aid of pins.

The limb was now laid carefully in an *empty* fracture box, a little cotton placed under the heel, and the foot tied to the foot board, where it was allowed to remain for four days, at the expiration of which period the whole was dry and hard, the limb being cased as firmly as in plaster.

The patient was then allowed to remain in bed without any other dressing except the splints; and on the ninth of January, thirteen days after the injury, a bandage was doubled around his neck, carried down behind the calf of the leg, then in front of the ankle, over the instep, and round under the foot to the instep again so as to form a sling to raise the foot a little from the ground and he was allowed to walk about with crutches. In this way he continued until February 7th, when the apparatus was taken off, before the class, the limb being perfectly straight and firm, and without the slightest deformity; and on the 13th of February, seven weeks after the injury, the man was discharged. In this instance, the apparatus was not touched until the fourth week, when a simple roller was applied to tighten it, owing to looseness consequent on the shrinking of the muscles. One of the objections raised to the use of the apparatus was thus readily obviated without injury to the patient; for, as the splints did not meet before and behind the leg, it was easy to fold the surplus bandage in, without causing any welt on the skin, while the bandage, having been previously washed, shrunk but little.

Case second.—*Fracture of the fibula two inches above the joint—Application of the apparatus seventeen days after the injury—Cure without deformity.*

Patrick D——, æt. 42 years, a labourer, fell off a step on the 15th of January, and fractured his fibula obliquely, two inches above the external malledus. Owing to the inflammation, leeches, and the antiphlogistic course, with the use of the fracture box, were continued until February 1st, seventeen days after the accident, when the apparatus was applied as in the preceding case; except that the splints were continued under the bottom of the foot, being slit up so that the fold under the foot did not interfere with the application of the splint to the sole, thus preventing all motion at the ankle joint. After the apparatus had been dried in the fracture box, with the foot *well turned in*, for five days, the patient was allowed to walk about, and on the 10th of February, twenty six days after the accident, he walked up to the third story of the house, and was operated on by Dr. Harris for cataract. On February 21st, the apparatus was removed—there being not the least deformity perceptible even to the touch.

Case third.—*Oblique fracture of both bones of the leg—Application of the apparatus nine days after the accident—Cured.*

Patrick C——, æt. 23 years, a laborer, whilst working on a rail road on the 18th of January, was knocked down by the caving in of a bank of earth, and both bones of his leg were broken obliquely, near the middle. He was treated in the usual way by the fracture box, until the 27th of January, when the starch dressing was applied. January 31st, four days afterwards, was allowed to rise and walk, by degrees, more each day, until February 25th, thirty eight days after the accident, when the apparatus was removed. The limb was perfectly straight without any motion between the bones, and strong enough to allow him to walk upon it, but reduced from a large size to nearly nothing, by the pressure and perfect rest. In this instance, the apparatus was not touched until the sixth day after its application, when on his complaining of its tightness over the instep, the foot was soaked for a few minutes in hot water, and, by introducing a spatula under the bandage, it was raised sufficiently to free the point of pain. Being then allowed to harden, he suffered no inconvenience afterwards.

The next three cases were of fractures of the thigh, in which, as there was but the one bone to act on, and other objects to be considered than the mere apposition of the fractured ends, it was applied at first, on a more advanced stage.

Case fourth.—*Oblique fracture of the middle of the femur—Application of the apparatus fifty-three days after the accident—Cure without deformity.*

Francis McG——, æt. 22 years, of good habits, fell on the 22d of November, down the hatchway of a vessel and fractured his clavicle and femur. The clavicle was dressed with the usual apparatus, and the femur treated by the long fracture box, fastened on the double inclined plane, until January 14th, fifty-three days after the injury, when the union not being firm, although there was considerable bony deposition, the apparatus was applied as follows:—A roller was carried smoothly up from below the knee to the groin, the limb being held up and extended by assistants; starched as in the first case, and covered by a second roller. A long splint of binder's board was then applied, from the tuberosity of the ischium to below the knee, or the back part of the thigh, and another from the groin to the patella, in front—so as to surround the limb entirely, except for the space mentioned in the dressing of the leg. These were then covered in the same manner as the splints in the first case, and a simple roller applied from the toes up to the lower part of the starch, so that it could be renewed at pleasure. The limb was laid on a simple inclined plane, until the apparatus dried. Five days were necessary to dry it, and the man was then allowed to walk about, the limb being supported by the sling before mentioned, the splint behind preventing all flexion at the knee. On the 2d of February, about ten weeks after the accident, the apparatus was removed, without there being found any deformity or perceptible shortening, the measurement showed it to be a little more than a quarter of an inch less than the sound limb, and on the 7th of February, the patient left the hospital.

CASES FIFTH and SIXTH.—*Oblique fracture of the upper third of the femur—Application of the apparatus thirty days after the injury—Perfect cure.*

Thomas H——, æt. 26, a labourer, fractured his thigh at its upper third, December 6th, about fifty miles from town. He was dressed in the neighbourhood, and did not arrive at the hospital, till the third day after the accident, owing to the destruction of part of the rail road. The limb was much inflamed and swollen, and was treated at first by the inclined fracture box, lotions, &c., until January 6th, when the apparatus was applied to it, and dried in the same manner as in the preceding case. On the 14th of January, the man was allowed to walk about, and the apparatus remained untouched, till its removal, February 12th, there being perfect union, and only one-eighth of an inch shortening by close measurement, and none perceptible in his gait; and on the 21st of February, eleven weeks after the injury, he was discharged.

The same apparatus was applied to Patrick E——, (who was admitted February 6th, with an oblique fracture, caused by blasting,) on the 19th of February, thirteen days after the accident, and enabled him to sit up in bed five days afterwards, and on February 25th, to walk the length of the room. On his standing up, he feels too weak to walk readily, but has every prospect of doing so shortly. At present, he complains of no inconvenience from the dressing, and is able to turn about in his bed; the limb being but little shortened by measurement over the splints. The termination of the case will be reported hereafter.

The advantages to be derived from an apparatus of the kind described, are too evident to need enumeration. In the case of fractures of the leg, it enables the patient to move about in fifteen days, with perfect safety. It has not been deemed expedient to apply it so early as M. Velpeau has done, owing to the severe contusions which complicate most of the fractures received here; but, with this restriction it might, as far as the experience of these cases prove, be used in all simple fractures, as few will be found, in private practice, more severe than those on which it has been tried. In respect to fractures of the thigh, the application cannot be made quite so early, as the contractile power of the muscles ought previously to be overcome by some apparatus for making extension. After this, however, the bandage prevents this action, and the weight of the limb in the erect posture, prevents shortening. In hospital practice, it also promises to be of great utility, by doing away the risk of sloughs on the sacrum, from the constant pressure consequent on the long confinement on the back, and adds very materially to the patient's comfort, by allowing him to rise to a window, or to go from one apartment to another. In case second, it enabled a man to rise and undergo an operation for cataract, in a place where the light was better than in his own room. In the last case reported, sufficient time has not elapsed, to prove the success of the dressing, when applied *early* in cases of fracture of the thigh; but should it succeed, as there is at present every prospect of its doing, it will remedy what has lately proved a great objection to the old method of treatment, namely, sloughing of the heel and its terrible consequences.

